# The Emerging Potential of Diabetes Technology to Improve Metabolic Dysfunction-Associated Steatotic Liver Disease in Patients with Type 1 Diabetes: A Narrative Review

**DOI:** 10.3390/biomedicines14091986

**Published:** 2026-09-03

**Authors:** Anna Garmpi, Vaia Lambadiari, Chrysi Koliaki

**Affiliations:** 1 First Propaedeutic Department of Internal Medicine and Diabetes Center, Laiko General Hospital, Medical School, National and Kapodistrian University of Athens, 17 Agiou Thoma Street, 11527 Athens, Greece; annagar@windowslive.com; 2 Research Unit and Diabetes Center, Second Propaedeutic Department of Internal Medicine, Attikon University Hospital, Medical School, National and Kapodistrian University of Athens, 1 Rimini Street, 12462 Athens, Greece; vlambadiari@gmail.com

**Keywords:** automated insulin delivery, continuous glucose monitoring, diabetes technology, hepatic steatosis, hepatic fibrosis, insulin resistance, insulin pump, metabolic dysfunction-associated steatotic liver disease, type 1 diabetes

## Abstract

Metabolic dysfunction-associated steatotic liver disease (MASLD) has emerged as an increasingly common comorbidity in individuals with type 1 diabetes (T1D), driven by the rising prevalence of obesity and insulin resistance in this population. Modern diabetes technologies have transformed T1D management and may favorably influence several pathophysiological pathways implicated in T1D-associated MASLD. We aimed to summarize and critically appraise current evidence regarding the emerging potential of diabetes technologies, such as continuous glucose monitoring (CGM), continuous subcutaneous insulin infusion (CSII) and automated insulin delivery (AID) systems, to influence MASLD-related outcomes in adults with T1D, and further discuss therapeutic implications and remaining evidence gaps. The existing evidence remains limited and is predominantly based on observational cross-sectional studies. The available data suggest that diabetes technologies may favorably influence the metabolic milieu associated with MASLD by increasing time in range, reducing glycemic variability and hypoglycemia, optimizing insulin delivery and improving the overall metabolic control. However, evidence for an association with hepatic outcomes remains limited, indirect and inconsistent. Emerging observational data have linked less favorable CGM-derived metrics, particularly lower time in range and greater glycemic variability, with hepatic steatosis and fibrosis, although some studies have identified insulin resistance as a stronger determinant than glycemic metrics alone. CSII therapy has been associated with favorable metabolic profiles, but confounding factors may limit causal interpretation. No study has demonstrated yet a direct beneficial effect of AID systems on hepatic steatosis or fibrosis in patients with T1D. In conclusion, diabetes technologies may favorably influence several pathophysiological pathways involved in T1D-associated MASLD. However, current evidence regarding potential hepatic benefits remains limited and indirect, precluding technology-specific recommendations for MASLD prevention or treatment in T1D. Adequately powered, prospective randomized studies with standardized imaging-based hepatic outcomes are needed to establish causality and define evidence-based clinical recommendations.

## 1. Introduction

Metabolic dysfunction-associated steatotic liver disease (MASLD), formerly known as non-alcoholic fatty liver disease (NAFLD) [1], is currently the most common chronic liver disease [2]. Its global prevalence has continuously increased during the past decades, with recent estimates approaching 38% [3,4]. MASLD represents a systemic metabolic disease with serious and potentially life-threatening hepatic and extrahepatic complications [5]. Approximately 10–30% of patients with simple hepatic steatosis are expected to progress to metabolic dysfunction-associated steatohepatitis (MASH) and advanced liver disease, including fibrosis, cirrhosis and hepatocellular carcinoma [6,7]. The presence of MASLD, and especially hepatic fibrosis, has been associated with an excess risk of developing T2D, cardiovascular disease, chronic kidney disease and extrahepatic malignancies [5,8,9]. Recent large-scale real-world studies further support these associations, reporting an increased risk of incident heart failure and chronic kidney disease among individuals with MASLD compared with those without MASLD [10,11]. The highest prevalence of MASLD is reported in people with obesity (75%) [12] or T2D (69%) [7]. Obesity and metabolic abnormalities appear to interact in determining the risk and progression of MASLD [13].

Traditionally, MASLD has been linked to components of metabolic syndrome such as hyperglycemia, dyslipidemia, abdominal obesity and insulin resistance and shares common pathophysiological mechanisms with T2D [9]. Type 1 diabetes (T1D), an autoimmune disease characterized by absolute insulin deficiency due to pancreatic β-cell destruction, has historically been viewed as metabolically distinct from T2D. However, it is becoming increasingly evident that individuals with T1D are not free from metabolic syndrome manifestations. They often display obesity, insulin resistance and MASLD, which is the hepatic manifestation of metabolic syndrome [14].

The burden of MASLD in the T1D population and the associated clinical implications remain inadequately characterized and merit further investigation. Patients with T1D are increasingly affected by overweight and obesity, as a result of unhealthy lifestyle habits such as poor diet and physical inactivity, as well as peripheral hyperinsulinemia caused by the exogenous subcutaneous insulin administration [14]. The reported prevalence of MASLD in T1D varies widely depending on the diagnostic approach. Furthermore, the observed variability in prevalence estimates may reflect MASLD underdiagnosis in subjects with T1D, possibly related to the historical focus on MASLD in the context of T2D. According to the meta-analysis by De Vries et al., the overall pooled prevalence of MASLD among adults with T1D was reported to be 22.0% [15]. It should be noted that there was a significant variation depending on the diagnostic method applied, with abdominal ultrasound yielding higher MASLD detection rates compared to magnetic resonance imaging (MRI) [15]. The most updated epidemiological evidence on MASLD prevalence in T1DM is provided by a systematic review and meta-analysis including 13,000 adults with T1D, which reported a pooled prevalence of 22.2% with significant heterogeneity across individual studies [16]. Similar to the previous meta-analysis [15], the reported estimates varied substantially according to the diagnostic modality used, ranging from 9.8% with MRI to 57.0% with the non-invasive Hepatic Steatosis Index (HSI). This wide range of prevalence estimates is largely driven by differences in the diagnostic modalities used to identify MASLD, rather than reflecting true differences in disease prevalence across T1D populations. Importantly, there was a strong association of MASLD with obesity, with 82.3% of obese individuals with T1D having MASLD compared with 13.7% of the non-obese, highlighting the major contribution of excess adiposity to the MASLD burden in this population [16].

The above epidemiological observations clearly suggest that MASLD should no longer be viewed as an uncommon comorbidity in T1D and underscore the need to better characterize the mechanisms driving hepatic fat accumulation in this population. MASLD pathophysiology in T1D extends well beyond chronic hyperglycemia. The major proposed contributors comprise insulin resistance, portal insulin deficiency, peripheral hyperinsulinemia due to subcutaneous insulin injection, reduced hepatic insulin clearance, increased hepatic de novo lipogenesis, obesity and diabetic dyslipidemia [14]. Furthermore, the increased glycemic variability may contribute to oxidative stress, inflammation and lipotoxicity, thereby promoting hepatic fat accumulation and disease progression [14].

The clinical implications of MASLD diagnosis in individuals with T1D are not limited to hepatic fat accumulation. Emerging evidence suggests that MASLD is associated with an increased risk of both hepatic and extrahepatic complications in subjects with T1D, supporting its recognition as a systemic metabolic disorder rather than an isolated liver disease [17]. In adults with T1D, MASLD has been independently associated with an increased prevalence of atherosclerotic cardiovascular disease and impaired vascular function, ultimately leading to a markedly higher estimated 10-year cardiovascular risk, particularly in the presence of significant liver fibrosis [18,19,20,21]. Furthermore, MASLD with fibrosis has been linked to a higher prevalence of chronic kidney disease, whereas MASLD without fibrosis has been associated with diabetic retinopathy in the T1D population [22]. Based on these findings, MASLD emerges as a clinically relevant marker of adverse cardiometabolic health in T1D, although existing evidence is largely observational and does not establish causality [20].

In the past decades, diabetes technology has literally transformed the landscape of T1D management. Continuous glucose monitoring (CGM), continuous subcutaneous insulin infusion (CSII) and automated insulin delivery (AID) systems have been consistently associated with improved glycemic control, as shown by an increased time in range, reduced hypoglycemia and less pronounced glycemic variability [23]. Considering that these technologies may target and favorably influence several metabolic pathways beyond just improving glycated hemoglobin (HbA1c), they may also have the potential to influence the risk and severity of MASLD. However, evidence specifically addressing the relationship between diabetes technology and MASLD in T1D remains limited.

The aim of the present narrative review is to summarize the existing evidence on the effects of diabetes technologies, including CGM, CSII and AID systems, on MASLD-related outcomes in subjects with T1D. We briefly review the pathophysiological links between T1D and MASLD, provide an updated summary of the available data on the effect of diabetes technologies on metabolic and hepatic outcomes in the context of T1D, and highlight the potential role of insulin resistance, glycemic control and glycemic variability in shaping the hepatic phenotype of T1D. We finally critically assess whether the potential hepatic benefits of modern diabetes technologies are supported by direct or indirect clinical evidence.

## 2. Literature Search and Review Criteria

A comprehensive literature search was conducted in PubMed/MEDLINE, Embase, and Scopus from database inception to 31 July 2026. The search strategy combined controlled vocabulary and free-text terms related to three main concepts: type 1 diabetes, diabetes technology—including continuous glucose monitoring, continuous subcutaneous insulin infusion, insulin pump therapy and automated insulin delivery systems—and metabolic dysfunction-associated steatotic liver disease, including the previously used terms non-alcoholic fatty liver disease, hepatic steatosis, steatohepatitis and hepatic fibrosis.

Studies were considered eligible if they were original human studies conducted in adults (≥18 years) with T1D, or in mixed populations for which T1D-specific data could be separately extracted; evaluated CGM, CSII/insulin pump therapy, or AID as an exposure or intervention; and reported at least one MASLD-related hepatic outcome, including hepatic steatosis, MASLD/NAFLD, hepatic fibrosis, liver stiffness, controlled attenuation parameter (CAP), or validated non-invasive indices such as the Fatty Liver Index (FLI), Hepatic Steatosis Index (HSI), or fibrosis scores. Only full-text articles published in English were considered eligible.

Studies were excluded if they were conducted exclusively in pediatric populations; did not provide separately extractable T1D data; did not evaluate an eligible diabetes technology; did not report a MASLD-related outcome; or were reviews, editorials, commentaries, letters, conference abstracts, study protocols, case reports, or preclinical studies. Studies evaluating glycemic control, insulin resistance, or other metabolic parameters in relation to MASLD without directly assessing an eligible diabetes technology were not included in the direct evidence synthesis, although relevant findings from such studies were considered in the pathophysiological discussion of the review.

Following database searches, records were merged and duplicate records were removed using bibliographic identifiers and normalized title matching. Titles and abstracts were screened for relevance, followed by full-text assessment of potentially eligible reports against the predefined inclusion and exclusion criteria. Reference lists of relevant articles were also examined to identify potentially eligible publications.

**Figure 1:**
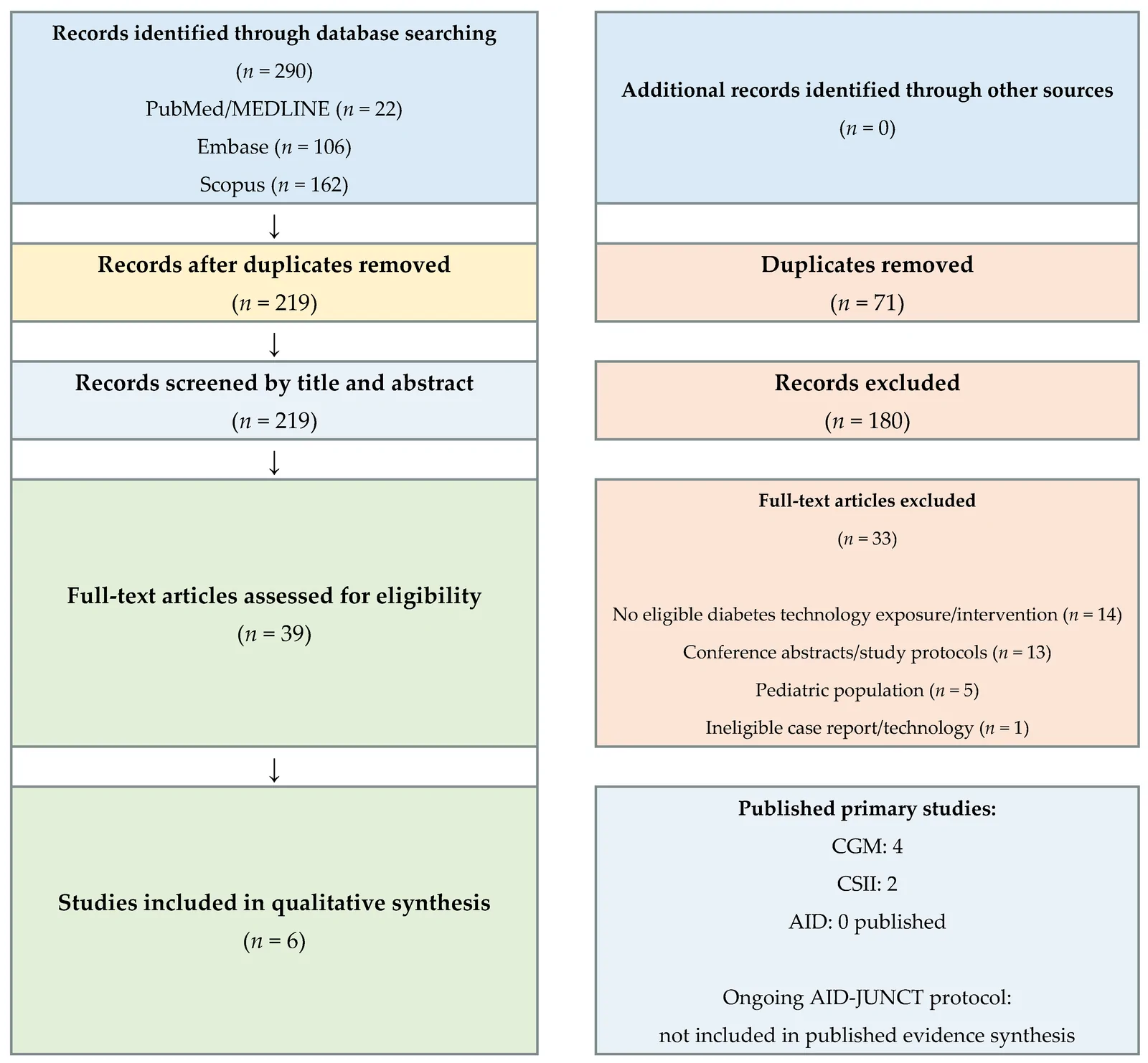
PRISMA-style flow diagram of study selection.

The database search identified 290 records (PubMed/MEDLINE, *n* = 22; Embase, *n* = 106; Scopus, *n* = 162). After removal of 71 duplicate records, 219 records were screened by title and abstract, of which 180 were excluded. Thirty-nine full-text articles were subsequently assessed for eligibility. Thirty-three articles were excluded, primarily because they did not evaluate an eligible diabetes technology (*n* = 14), were conference abstracts or study protocols (*n* = 13), involved pediatric populations (*n* = 5), or represented an ineligible case report/technology (*n* = 1). Six published studies fulfilled all eligibility criteria and were included in the qualitative synthesis. The detailed study selection process is summarized in a PRISMA-style flow diagram (Figure 1). Considering that the present manuscript is a narrative review, the flow diagram is intended to provide transparency regarding the literature search and study selection process rather than to imply that the review constitutes a systematic review.

## 3. Pathophysiological Mechanisms Underlying the Association Between T1D and MASLD

In individuals with T1D, hepatic steatosis develops when hepatic lipid influx exceeds disposal, resulting in excessive intrahepatic triglyceride accumulation. This imbalance may arise from increased de novo lipogenesis, enhanced free fatty acid (FFA) delivery from adipose tissue as a result of increased lipolysis, impaired fatty acid oxidation, and reduced triglyceride export through very low-density lipoprotein particles (VLDL). Collectively, increased adipose tissue lipolysis, enhanced intrahepatic triglyceride synthesis and impaired hepatic lipid disposal may all promote hepatic fat accumulation.

Importantly, the level of evidence supporting these mechanisms is heterogeneous. While several metabolic and physiological abnormalities have been directly demonstrated in individuals with T1D, their specific contribution to MASLD development has not been established to the same extent. For some downstream molecular pathways, the evidence is derived predominantly from experimental studies or extrapolation from other insulin-resistant metabolic settings. These distinctions should be considered when interpreting the proposed pathophysiological framework. Insulin resistance is now recognized as an important pathophysiological component of T1D and may contribute to both hepatic and cardiometabolic adverse outcomes [24]. Beyond lifestyle-related obesity, chronic peripheral hyperinsulinemia induced by subcutaneous insulin therapy may promote insulin resistance by downregulating insulin receptor expression and impairing insulin signaling in target tissues, including liver, skeletal muscle, adipose tissue and the vascular endothelium [24]. At the hepatic level, insulin resistance leads to increased endogenous glucose production, while in skeletal muscle it reduces glucose uptake, utilization and glycogen synthesis. In adipose tissue, defective insulin action stimulates lipolysis, resulting in an increased release of FFAs, glycerol and proinflammatory cytokines into the circulation. The increased FFA flux to the liver provides substrates for hepatic de novo lipogenesis and triglyceride accumulation, thereby contributing to MASLD development. In addition, vascular insulin resistance impairs blood flow to skeletal muscle and adipose tissue, further reducing peripheral glucose disposal and aggravating hyperglycemia. Collectively, these alterations form a vicious cycle that interlinks insulin resistance, hyperglycemia, dyslipidemia, inflammation and hepatic steatosis in people with T1D [24].

Mitochondrial dysfunction represents another potential significant mechanism linking T1D and MASLD. T1D-specific human data provide some support for alterations in hepatic energy metabolism; reduced hepatic ATP concentrations have been demonstrated in individuals with T1D, while recent metabolomic data in individuals with T1D and MASLD suggest perturbations in tricarboxylic acid cycle activity [25,26]. However, the direct contribution of mitochondrial dysfunction to MASLD development and progression in T1D remains incompletely established. Mitochondria are central regulators of metabolic flexibility, controlling cellular energy metabolism and fatty acid β-oxidation, and thereby determining the ability of hepatocytes to cope with an increased glucose and FFA availability [27]. Chronic hyperglycemia, recurrent hypoglycemia, and increased glycemic variability may impair mitochondrial structure and oxidative phosphorylation capacity, resulting ultimately in mitochondrial dysfunction, which is strongly associated with insulin resistance [28]. Insulin resistance leads to increased lipolysis in adipose tissue and increases the FFA flux to the liver, saturating mitochondrial oxidative capacity and thereby producing excessive reactive oxygen species (ROS). The oxidative stress may in turn trigger inflammation, hepatocellular damage and worsening of insulin sensitivity, thus establishing a vicious cycle that perpetuates itself and promotes hepatic fat accumulation and MASLD progression in patients with T1D [29].

Chronic hyperglycemia may directly contribute to hepatic steatosis in poorly controlled T1D patients. Insulin-independent glucose transport into hepatocytes occurs via glucose transporter 2 (GLUT2), whose hepatic expression is upregulated in the setting of hyperglycemia. When intracellular glucose availability exceeds the rate of oxidative metabolism and glycogen synthesis, excess glucose is diverted to lipogenic pathways through intermediates such as pyruvate and acetyl-CoA, leading to increased hepatic de novo lipogenesis. Thus, chronic hyperglycemia may provide excess substrate for hepatic lipid synthesis and promote the conversion of excess glucose into fat. Clinical evidence suggests that chronic hyperglycemia is associated with MASLD in T1D. In a cross-sectional study in 659 young adults with T1D, Della Pepa et al. showed that patients with HbA1c > 7.6% had significantly higher values of Fatty Liver Index (FLI) and HSI, compared to those with better glycemic control, with the association persisting after accounting for obesity status [30]. These findings suggest a potential contribution of chronic hyperglycemia to hepatic steatosis; however, its independence from adiposity requires further confirmation [30]. Moreover, the combined presence of hyperglycemia and hyperinsulinemia may activate key transcription factors involved in hepatic lipid metabolism, particularly sterol regulatory element binding proteins (SREBPs) and carbohydrate-responsive element binding protein (ChREBP). These transcription factors regulate the expression of a multitude of genes involved in the uptake and synthesis of cholesterol, fatty acids, triglycerides and phospholipids. SREBPs and ChREBP also induce expression of lipogenic enzymes such as liver-type pyruvate kinase, fatty acid synthase, and acetyl-CoA carboxylase, further promoting hepatic de novo lipogenesis and triglyceride accumulation [31,32].

Hypoglycemia can also indirectly increase the risk of MASLD in patients with T1D. Fast-acting carbohydrates are often required to treat hypoglycemia in people with T1D, and the fear of recurrent hypoglycemia often promotes defensive snacking and overeating. Over time, these behaviors could lead to progressive weight gain, increased insulin requirements, worsening insulin resistance and increased glycemic variability. Moreover, the repeated consumption of fructose-containing foods and beverages used to correct hypoglycemia may further stimulate hepatic de novo lipogenesis [31].

Altered insulin kinetics represent a biologically plausible mechanism that may contribute to MASLD development in T1D. Human studies in individuals with T1D comparing intraportal with subcutaneous insulin administration support the non-physiological insulin exposure associated with peripheral insulin delivery [33]. Under physiological conditions, endogenous insulin is secreted into the portal circulation, where approximately 50–80% is extracted by the liver during first-pass metabolism through receptor-mediated uptake and degradation by insulin-degrading enzyme (IDE). In contrast, exogenous insulin administered subcutaneously bypasses the portal circulation and abolishes the physiological pulsatile delivery of insulin to the liver, resulting in relative portal hypoinsulinemia in parallel with peripheral hyperinsulinemia. Chronic hyperinsulinemia may also reduce hepatic insulin clearance, further increasing systemic insulin exposure. These changes collectively impair hepatic insulin signaling, reduce suppression of hepatic glucose production, promote hepatic lipogenesis, and favor intrahepatic triglyceride accumulation, contributing thus to MASLD development in people with T1D [31,32,34].

The diabetic dyslipidemia in T1D further promotes fat accumulation in the liver. Insulin resistance and peripheral hyperinsulinemia stimulate lipolysis in adipose tissue, leading to an excess of circulating FFAs delivered to the liver [29,34]. At the same time, hepatic fatty acid oxidation and triglyceride export via VLDL are impaired, promoting intrahepatic lipid retention. Ectopic lipid deposition is the result of excess delivery of FFAs, and adipose tissue dysfunction is characterized by decreased secretion of adiponectin and increased production of proinflammatory adipokines that in turn perpetuate chronic inflammation, exacerbate insulin resistance, and promote hepatic steatosis [31,32].

Other hormonal abnormalities in T1D may also contribute to MASLD pathogenesis. T1D is characterized by fasting and postprandial hyperglucagonemia, due primarily to loss of inhibition of α-cell glucagon secretion by endogenous insulin within the islets. Dysregulated postprandial glucagon secretion has been directly demonstrated in human studies of individuals with T1D [35]. However, its specific contribution to MASLD development remains uncertain. Glucagon normally stimulates hepatic glucose production via glycogenolysis and gluconeogenesis and also promotes fatty acid oxidation, thus decreasing hepatic lipid accumulation. The coexistence of hyperglucagonemia and hepatic steatosis in T1D has therefore raised the hypothesis of hepatic glucagon resistance, particularly with respect to lipid metabolism. However, direct evidence for hepatic glucagon resistance in individuals with T1D is currently absent, and this concept should be regarded as a biologically plausible but largely untested mechanism rather than an established feature of T1D-associated MASLD. Hyperglucagonemia may be further aggravated by amylin deficiency, as amylin is co-secreted with insulin by pancreatic β-cells and normally suppresses postprandial glucagon secretion [31,32,34].

In summary, MASLD associated with T1D represents the integration of classical metabolic disturbances and disease-specific processes related to exogenous insulin treatment. Hepatic lipid accumulation and disease progression are promoted by the convergence of insulin resistance, mitochondrial dysfunction, chronic hyperglycemia, recurrent hypoglycemia, altered insulin kinetics, dyslipidemia, and aberrant glucagon signaling. Importantly, several of these pathophysiological pathways (hyperglycemia, peripheral hyperinsulinemia, hypoglycemia) are potentially modifiable by modern diabetes technologies, as analytically discussed in the sections below.

## 4. Brief Overview of Diabetes Technologies and Their Clinical Benefits in T1D

Over the last two decades, diabetes technology has profoundly transformed the management approach of T1D, shifting care from conventional glucose monitoring and insulin administration toward more personalized, data-driven treatment adjustment. The main diabetes technologies currently used in T1D include CGM, CSII and AID systems, as well as digital health tools such as mobile applications [23].

CGM systems are now a core component of T1D management, providing real-time or intermittently scanned measurements of interstitial glucose concentrations and enabling comprehensive assessment of glycemic control beyond HbA1c through metrics such as time in range (TIR), time above range (TAR), time below range (TBR) and glycemic variability [23,36]. Compared with conventional self-monitoring of blood glucose, CGM use has been associated with improved glycemic control and reduced hypoglycemia burden [36]. Importantly, CGM-derived metrics provide information on glucose excursions and short-term variability that is not captured by HbA1c alone.

CSII delivers rapid-acting insulin through a subcutaneous pump, allowing flexible adjustment of basal insulin delivery and meal-related boluses according to glucose trends, carbohydrate intake and physical activity [23]. Compared with multiple daily injections (MDI), CSII has been associated with improvements in glycemic control, reduced glycemic variability and lower rates of severe hypoglycemia in individuals with T1D [23]. Moreover, pump therapy may allow more individualized insulin delivery and, in some patients, reduce total daily insulin requirements, which could be relevant for metabolic outcomes beyond glycemia.

The most advanced form of diabetes technology is currently AID systems, mainly represented by hybrid closed-loop systems [37]. These systems combine CGM data with insulin pump therapy via algorithms that automatically adjust insulin delivery according to real-time glucose data [23,38,39]. These systems have shown a consistent ability to increase TIR, decrease both TAR and TBR, and improve overall glycemic stability versus conventional insulin therapy through continuous adjustment of insulin delivery based on changing glucose levels.

Digital health tools, including telemedicine platforms and mobile applications, are increasingly being used as adjuncts to core diabetes technology. They facilitate remote monitoring, structured interpretation of glucose data, behavioral feedback, and shared decision-making between individuals with T1D and healthcare professionals. Importantly, they may support adherence and lifestyle modifications as well as enable earlier therapeutic adjustments [40,41,42,43].

Collectively, current clinical practice guidelines recommend early consideration of CGM, CSII and AID systems according to individual needs and preferences, recognizing AID systems as the preferred insulin delivery modality for many individuals with T1D, in the context of glycemic management [23,44]. These recommendations are based on established glycemic benefits and are not currently informed by hepatic or MASLD-specific outcome data. Importantly, these technologies have shifted the therapeutic focus in T1D management from HbA1c-centered care toward a broader evaluation of glycemic control, including TIR, glycemic variability and hypoglycemia exposure. These benefits may have implications beyond traditional glycemic endpoints, raising the possibility that advanced diabetes technologies could also influence broader metabolic complications, including MASLD.

## 5. Potential Mechanisms Linking Diabetes Technology to MASLD Presence and Severity

While direct evidence linking diabetes technology to improvements in MASLD in the setting of T1D is currently limited and completely absent for AID systems, several biological and metabolic pathways provide a hypothesis-generating framework through which contemporary diabetes technologies could potentially influence hepatic steatosis and MASLD progression in people with T1D.

One potential mechanism is improved glycemic stability. Recurrent glucose excursions have been linked with oxidative stress, mitochondrial dysfunction, endothelial injury and activation of inflammatory pathways, which all lead to hepatic lipid accumulation [29,31,32]. CGM-guided insulin therapy and AID systems improve glycemic stability by reducing both hyperglycemic and hypoglycemic excursions and could theoretically reduce metabolic stress relevant to MASLD [23,36,37,38].

The increased TIR may be another important mediator of hepatic benefit. Higher TIR reflects more time in near-normoglycemia and less time in both hyperglycemia and hypoglycemia. Therefore, sustained improvements in TIR may limit glucose-driven hepatic de novo lipogenesis and reduce chronic metabolic stress in hepatocytes.

Another possible mechanism is the optimization of insulin delivery. CSII and AID systems provide more adaptive and responsive subcutaneous insulin delivery than fixed basal-bolus regimens by allowing programmable basal rates and algorithm-guided adjustments based on real-time glucose data. However, they fail to restore physiological portal insulin delivery or first-pass hepatic insulin exposure. More precise insulin delivery may reduce overall insulin exposure and, in some individuals, lower total daily insulin requirements, which could theoretically attenuate insulin-mediated lipogenesis and other metabolic consequences of chronic hyperinsulinemia [24].

Modern diabetes technologies may also reduce the burden of hypoglycemia, limiting compensatory overeating and allowing for healthier dietary behaviors, improved body weight management, and increased patient engagement through continuous glucose feedback. These behavioral adaptations together may further improve insulin sensitivity and indirectly reduce hepatic fat accumulation [31]. On the other hand, countervailing considerations should also be acknowledged, such as the clinical observation that reduced hypoglycemia may in some patients accompany weight gain, suggesting that the potential metabolic benefits of diabetes technology may not be uniform across all individuals.

**Figure 2:**
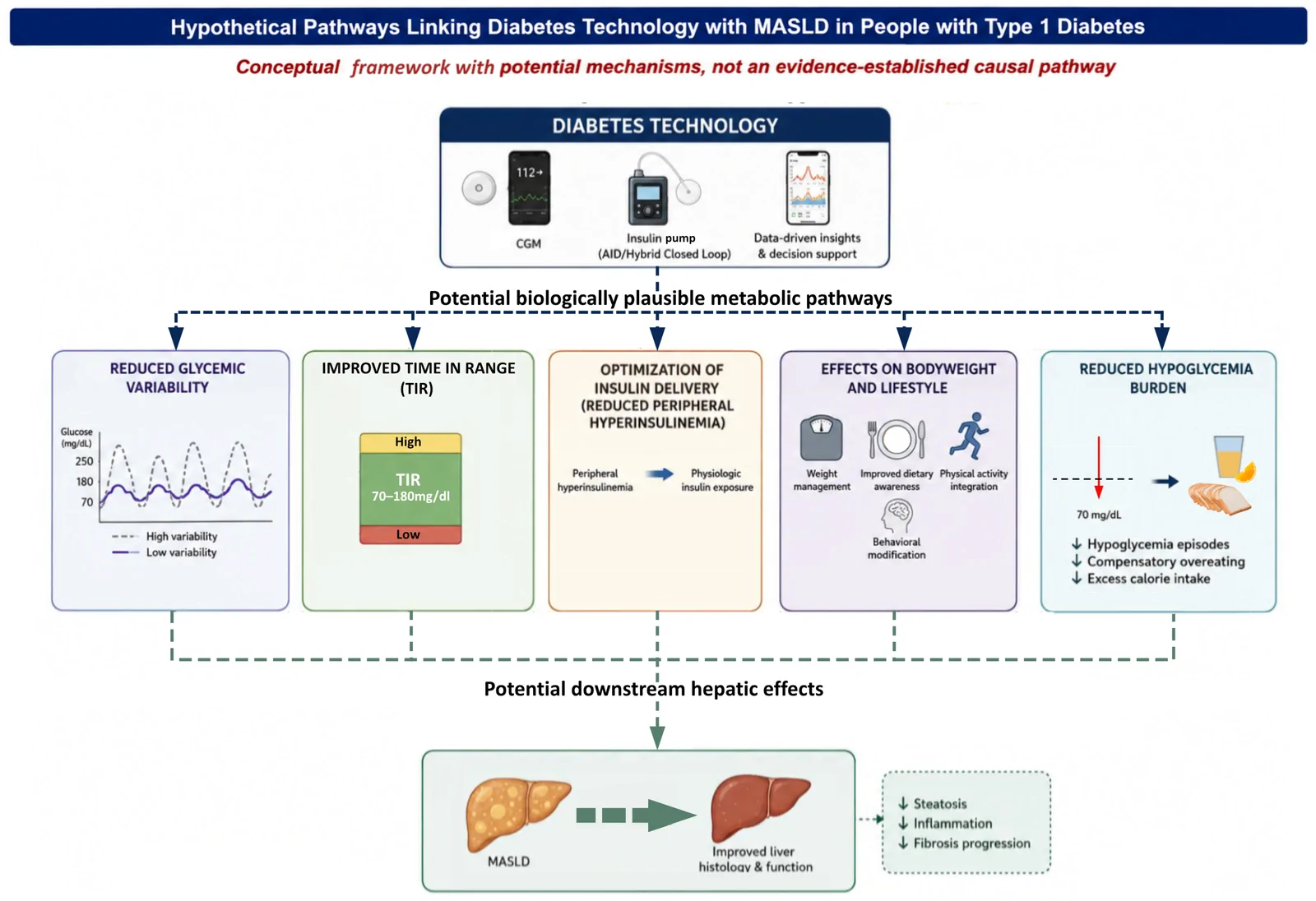
Hypothetical pathways linking diabetes technology with MASLD in people with type 1 diabetes. This schematic represents a conceptual model of potential biological and metabolic pathways through which diabetes technologies may indirectly influence MASLD-related processes in T1D. Dashed arrows indicate hypothetical or potential relationships and do not imply an established causal pathway. Improvements in glycemic stability, time in range, insulin delivery, hypoglycemia burden, and the broader metabolic milieu may theoretically attenuate processes involved in hepatic steatosis and MASLD progression, including de novo lipogenesis, oxidative stress, lipotoxicity, and inflammation. However, direct evidence that diabetes technology improves hepatic steatosis, fibrosis, or MASLD progression in T1D remains limited, indirect, and partly inconsistent. Dedicated prospective clinical trials with standardized liver-related endpoints are required to determine whether these potential metabolic effects translate into clinically meaningful hepatic benefits. Abbreviations: AID: automated insulin delivery; CGM: continuous glucose monitoring; MASLD: metabolic dysfunction-associated steatotic liver disease.

Taken together, these pathways provide a biological rationale for a potential effect of diabetes technologies on MASLD; however, they remain largely hypothetical and should not be interpreted as established causal mechanisms or evidence of direct hepatic benefit. Figure 2 illustrates these proposed, rather than proven, pathways, including better glycemic stability, optimized insulin delivery, lower overall insulin exposure, reduced burden of hypoglycemia, healthier lifestyle behaviors, and improved insulin sensitivity. Whether these indirect metabolic benefits translate into clinically meaningful improvements in hepatic steatosis or fibrosis remains to be established in well-designed prospective studies.

## 6. Evidence on CGM and MASLD Outcomes in T1D

CGM provides a detailed evaluation of glycemic control, supplementing HbA1c with indices such as TIR, TAR, TBR, glucose management indicator (GMI), and glycemic variability. These metrics have been recently investigated as potential markers of hepatic steatosis and fibrosis in T1D. Importantly, interpretation of the available evidence requires consideration of the method used to assess MASLD, as studies have employed either imaging-based modalities, including ultrasound and VCTE/CAP, or surrogate biomarker indices such as FLI and HSI, which may contribute to heterogeneity across findings.

The initial evidence linking CGM-derived metrics with MASLD in patients with T1D was provided by Aernouts et al., who reported that subjects with ultrasound-defined MASLD had lower TIR and TBR and higher TAR than those without MASLD. These metrics remained independently associated with MASLD after multivariable adjustment, whereas HbA1c did not differ significantly between groups [45].

Similarly, Mantovani et al. reported that TAR was independently associated with higher controlled attenuation parameter (CAP) values reflecting hepatic steatosis in 262 adults with T1D undergoing vibration-controlled transient elastography (VCTE) for MASLD assessment. This association persisted after adjustment for age, sex, body mass index (BMI), HbA1c, and total daily insulin dose, although BMI emerged as the strongest correlate of hepatic steatosis [46].

In contrast, Vergani et al. found no independent association between CGM-derived metrics and VCTE-defined MASLD in 198 adults with T1D. Rather, hepatic steatosis was independently associated with lower estimated glucose disposal rate (eGDR), suggesting that insulin resistance may be a stronger correlate of MASLD than short-term glycemic metrics [47].

Finally, Fedulovs et al. used CGM-based clustering to identify distinct glycemic phenotypes and found that participants with poorer glycemic control had lower estimated insulin sensitivity, higher FLI and HSI values, elevated liver enzymes and greater systemic inflammation. Although these data were exploratory and based on surrogate indices rather than imaging-confirmed MASLD, they suggest that integrated CGM-derived phenotypes may help identify individuals at increased metabolic and hepatic risk [48].

In summary, the available evidence supports an association between unfavorable CGM profiles and MASLD-related metabolic abnormalities in T1D. However, these findings remain partly inconsistent, likely owing to differences in study populations, methods used to assess hepatic steatosis, and CGM metrics evaluated. Since all available studies are cross-sectional, prospective longitudinal and interventional studies are needed to determine whether improving CGM-derived metrics through diabetes technology can prevent or attenuate MASLD progression.

## 7. Evidence on CSII Therapy and MASLD Outcomes in T1D

Compared with the growing evidence on CGM, studies specifically evaluating the association between CSII and MASLD in adults with T1D remain scarce. To date, only a few cross-sectional studies have addressed this question, and they suggest potentially favorable metabolic associations rather than a proven causal hepatic benefit. Similar methodological heterogeneity applies to the available CSII evidence, with MASLD assessed using either imaging-based methods or surrogate biomarker indices.

In the largest multicenter study including 1417 adults with T1D, Csermely et al. found that CSII users were younger and had better glycemic control than individuals treated with MDI. In this study, although MASLD without fibrosis occurred with comparable frequency in both groups, MASLD with significant fibrosis was less common among CSII users in unadjusted analyses. However, the association was no longer significant after adjustment for age, HbA1c and other potential confounders, suggesting that the observed difference was largely explained by different baseline group characteristics, particularly age [49]. Complementary findings were reported by Della Pepa et al., who studied 659 adults with T1D and found that CSII use was associated with lower FLI and HSI values, as well as lower total daily insulin dose, waist circumference, and triglyceride levels [50]. These differences were observed primarily among women and not men, raising the possibility of sex-specific metabolic associations. Notably, women using CSII also had a lower visceral adiposity index than those treated with MDI, and the authors proposed that lower peripheral insulin exposure within a more favorable hormonal and adipose-tissue milieu may partly explain this sex-specific finding. However, no formal power analysis for sex-stratified comparisons was reported; therefore, this observation should be considered hypothesis-generating and requires confirmation in larger studies specifically designed to evaluate sex-specific effects. Overall, the available evidence suggests that CSII may be associated with a more favorable metabolic profile relevant to MASLD but does not exert an independent protective effect on hepatic steatosis or fibrosis [49,50].

## 8. Evidence on AID Systems and MASLD Outcomes in T1D

Although AID systems have consistently demonstrated superior glycemic outcomes compared with other currently available diabetes technologies [51,52], no study has specifically evaluated their effects on hepatic steatosis, fibrosis or other MASLD-related outcomes in individuals with T1D. Consequently, any potential hepatic benefit remains speculative and must currently be inferred from the favorable metabolic effects of these systems rather than from direct clinical evidence. Nevertheless, given their well-documented potential to improve glycemic stability, reduce glycemic variability, minimize hypoglycemia and optimize insulin delivery, AID systems represent a promising platform for future studies investigating the potential effects of diabetes technology on MASLD. Prospective studies incorporating imaging-based assessment of hepatic steatosis and fibrosis are required to determine whether these metabolic improvements translate into clinically significant liver-related outcomes. In this context, it is important to refer to an ongoing prospective randomized clinical trial (RCT) in 42 adults with T1D, which aims to investigate whether adding tirzepatide to AID therapy vs. AID alone would confer additional glycemic benefits (AID-JUNCT trial, ClinicalTrials.gov ID: NCT06630585). In this trial, MASLD and liver fibrosis assessed by VCTE was used as an exploratory outcome. This is the first prospective RCT assessing MASLD in T1D patients under AID therapy, and its results are eagerly anticipated [53]. A broader search of clinical trial registries identified no additional ongoing or registered trials specifically evaluating the effects of AID systems on hepatic steatosis, fibrosis, or other MASLD-related outcomes in individuals with T1D. One additional registered study (NCT05933018) investigates the relationship between nocturnal hyperglycemia and hepatic steatosis using CGM, insulin-pump data, and liver elastography; however, it does not evaluate AID as an intervention. Thus, the current landscape of prospective research directly addressing AID and MASLD-related outcomes in T1D remains extremely limited.

Table 1 summarizes the key findings of the major studies investigating the effects of CGM, CSII and AID systems on metabolic and hepatic outcomes in patients with T1D [45,46,47,48,49,50,53].

## 9. Challenges and Limitations of Current Evidence

The current evidence base is limited by the small number of T1D-specific studies assessing liver outcomes in relation to diabetes technology. Most available data are observational and cross-sectional, which precludes causal inference. In addition, several conclusions are extrapolated from metabolic outcomes rather than from direct hepatic endpoints.

Another important limitation is the heterogeneity in MASLD definitions and diagnostic methods across studies, including ultrasound, VCTE/CAP, biomarker-based indices such as FLI and HSI, and fibrosis scores. These approaches differ in their ability to detect and quantify hepatic steatosis and fibrosis, while surrogate indices may also be influenced by metabolic variables incorporated into their calculation. Consequently, differences in MASLD assessment may contribute to variability in prevalence estimates and observed associations, limiting direct comparability across studies and the overall interpretation of the available evidence. Sample sizes are relatively small in most liver-focused studies, and follow-up is short or absent. Important confounders such as BMI, diet, and physical activity are difficult to control completely. Concurrent use of adjunctive pharmacotherapies represents an additional potential source of residual confounding. Although medication use was reported in several observational studies, treatment with agents such as GLP-1 receptor agonists, SGLT inhibitors, or metformin was not consistently accounted for in multivariable analyses and may have influenced metabolic and hepatic outcomes.

Finally, complete patient-level independence across the included observational studies could not be confirmed from the published data. Although some cohorts were clearly recruited from distinct geographical settings, several Italian multicenter studies shared participating centers and investigators, raising the possibility of partial patient overlap. Therefore, the six published studies should not necessarily be interpreted as representing six entirely independent patient populations, which may further limit the effective size of the available evidence base.

## 10. Clinical Implications

Although current evidence does not support the use of diabetes technology specifically as a treatment for MASLD, it has several important clinical implications. Screening for MASLD in T1D is not yet universally standardized, but it may be considered in selected high-risk patients, particularly in those with obesity, central adiposity, dyslipidemia, hypertension, long-standing suboptimal glycemic control, or features suggestive of insulin resistance [54]. Emerging T1D-specific data indicate that MASLD and liver fibrosis are associated with a broader adverse cardiometabolic profile and may coexist with micro- or macrovascular complications, supporting a more proactive hepatic risk assessment in vulnerable patients. In clinical practice, a pragmatic stepwise approach, informed by existing consensus recommendations for MASLD screening in people with diabetes [54] and adapted to the context of T1D, could include identification of high-risk phenotypes and routine liver biochemistry, followed by non-invasive fibrosis assessment or imaging when clinically indicated. Given the limited T1D-specific evidence, this approach should be regarded as a pragmatic clinical framework rather than a validated or T1D-specific guideline-endorsed screening algorithm.

Modern diabetes technologies should be viewed not only as tools for improving glycemic control but also as part of a broader strategy to optimize metabolic health. By increasing TIR, reducing glycemic variability and hypoglycemia, and optimizing insulin delivery, CGM, CSII, and AID systems may contribute to a more comprehensive approach to cardiometabolic risk reduction. However, any potential hepatic benefits should be considered indirect at present rather than firmly established.

The optimal management of MASLD in T1D requires a multidisciplinary approach involving diabetologists, dietitians, and, when appropriate, hepatologists. Combining lifestyle interventions with advanced diabetes technologies may maximize metabolic benefit and may facilitate the earlier identification of individuals with increased hepatic risk.

## 11. Future Directions

Future research should include prospective interventional studies specifically designed to evaluate the hepatic outcomes of diabetes technologies in T1D. These studies should incorporate standardized imaging-based assessment of hepatic steatosis and fibrosis, including VCTE, MRI and validated fibrosis scores, to determine if improvements in glycemia translate into clinically relevant benefits in the liver. Greater standardization of MASLD diagnostic criteria and outcome definitions is also warranted to improve comparability across cohorts.

In addition, artificial intelligence and machine-learning approaches have shown promise for MASLD detection and risk stratification in populations with diabetes [55]. However, their integration with digital biomarkers and advanced CGM-derived analytics for MASLD risk prediction specifically in individuals with T1D remains largely unexplored and should currently be regarded as a promising area for future research rather than an evidence-established approach. Future studies should also evaluate whether combining diabetes technologies with structured lifestyle interventions or pharmacological therapies provides additive benefits for both hepatic and cardiometabolic outcomes.

## 12. Summary and Concluding Remarks

MASLD is increasingly recognized as an important metabolic comorbidity in individuals with T1D, with insulin resistance, chronic hyperglycemia, altered insulin kinetics, and glycemic instability representing key links between the two conditions. Modern diabetes technologies, including CGM, CSII and AID systems, may favorably influence several of these pathways by improving glycemic stability, optimizing insulin delivery, reducing hypoglycemia, and enhancing overall metabolic control.

However, current evidence for a direct beneficial effect of diabetes technology on hepatic steatosis or fibrosis remains limited and largely indirect, and there is no direct causal evidence that diabetes technology improves MASLD in T1D. Available studies are limited, cross-sectional and heterogeneous with respect to study populations, MASLD assessment methods, and metabolic outcomes. Overall, diabetes technology represents a promising strategy for improving the metabolic milieu associated with MASLD in T1D, but dedicated prospective studies with standardized liver endpoints are required before causal conclusions can be drawn or specific liver-directed recommendations can be made.

## Figures and Tables

**Table 1:** Major studies investigating the effects of CGM, CSII and AID systems on metabolic and hepatic outcomes in patients with T1D.

First Author (Year of Publication) [Ref]	Diabetes Technology	Study Population	MASLD Assessment	Main Findings	Limitations
Aernouts, et al. (2024) [45]	CGM	302 adults with T1D	Ultrasound	MASLD was independently associated with lower TIR (*p* = 0.028), higher TAR (*p* = 0.007), and lower TBR (*p* = 0.036); HbA1c was not significantly different between patients with and without MASLD (*p* = 0.068).	Cross-sectional design Ultrasound-based diagnosis
Mantovani, et al. (2025) [46]	CGM	262 adults with T1D	VCTE with CAP	Higher TAR was independently associated with higher CAP values (coefficient: 1.037; 95% CI: 0.216–1.858; *p* = 0.013), after adjustment for age, sex, BMI, HbA1c, and total daily insulin dose. BMI was the strongest correlate of hepatic steatosis.	Cross-sectional design
Vergani, et al. (2025) [47]	CGM	198 adults with T1D	VCTE	No independent association was found between CGM metrics and MASLD. MASLD was independently associated with lower eGDR (beta coefficient = −0.367; 95% CI: −0.472 to −0.261; *p* < 0.001), after multivariable adjustment, suggesting a stronger role of insulin resistance.	Cross-sectional design
Fedulovs, et al. (2026) [48]	CGM	75 adults with T1D	FLI and HSI	Less favorable CGM-derived phenotypes were associated with lower insulin sensitivity (eGDR: 4.4 vs. 7.3 mg/kg/min; *p* = 0.001), higher FLI (29 vs. 17; *p* = 0.034), higher HSI values (37 vs. 35; *p* = 0.033), elevated liver enzymes, and greater systemic inflammation.	Exploratory clustering analysis Surrogate markers of MASLD rather than imaging
Csermely, et al. (2023) [49]	CSII vs. MDI	1417 adults with T1D	Biomarker-defined MASLD and fibrosis indices	MASLD with significant fibrosis was less common among CSII users in unadjusted analyses (odds ratio = 0.32; 95% CI: 0.14–0.70; *p* = 0.004); however, the association was no longer significant after multivariable adjustment.	Cross-sectional design
Pepa, et al. (2023) [50]	CSII vs. MDI	659 adults with T1D	FLI and HSI	CSII users had lower FLI (20.2 ± 21.2 vs. 24.8 ± 24.3; *p* = 0.003) and HSI (36.2 ± 4.4 vs. 37.4 ± 4.4; *p* = 0.003) compared with MDI users. Lower FLI and HSI values were observed among women (*p* = 0.009 and *p* = 0.033, respectively), but not among men (*p* = 0.676 and *p* = 0.131, respectively).	Cross-sectional design
NCT06630585 (AID-JUNCT trial) [53]	AID + tirzepatide vs. AID + standard care	42 adults with T1D	VCTE	Ongoing study, results not published	

Note: MASLD assessment methods vary substantially across studies and include imaging-based modalities and surrogate biomarker indices, which should be considered when comparing findings across studies. Abbreviations: AID: automated insulin delivery; BMI: body mass index; CAP: controlled attenuation parameter; CGM: continuous glucose monitoring; CI: confidence intervals; CSII: continuous subcutaneous insulin infusion; eGDR: estimated glucose disposal rate; FLI: Fatty Liver Index; HbA1c: glycated hemoglobin; HSI: Hepatic Steatosis Index; MASLD: metabolic dysfunction-associated steatotic liver disease; MDI: multiple daily injections; T1D: type 1 diabetes; TAR: time above range; TBR: time below range; TIR: time in range; VCTE: vibration-controlled transient elastography.

## Data Availability

No new data were created or analyzed in this study. Data sharing is not applicable to this article.

## Abbreviations

The following abbreviations are used in this manuscript:

AID	Automated Insulin Delivery
BMI	Body Mass Index
CAP	Controlled Attenuation Parameter
CGM	Continuous Glucose Monitoring
ChREBP	Carbohydrate-Responsive Element-Binding Protein
CSII	Continuous Subcutaneous Insulin Infusion
eGDR	Estimated Glucose Disposal Rate
FFA	Free Fatty Acid
FLI	Fatty Liver Index
GMI	Glucose Management Indicator
GLUT2	Glucose Transporter 2
HbA1c	Glycated Hemoglobin
HSI	Hepatic Steatosis Index
IDE	Insulin-Degrading Enzyme
MASH	Metabolic Dysfunction-Associated Steatohepatitis
MASLD	Metabolic Dysfunction-Associated Steatotic Liver Disease
MDI	Multiple Daily Injections
MRI	Magnetic Resonance Imaging
NAFLD	Non-Alcoholic Fatty Liver Disease
RCT	Randomized Clinical Trial
ROS	Reactive Oxygen Species
SREBPs	Sterol Regulatory Element-Binding Proteins
T1D	Type 1 Diabetes
T2D	Type 2 Diabetes
TAR	Time Above Range
TBR	Time Below Range
TIR	Time In Range
VCTE	Vibration-Controlled Transient Elastography
VLDL	Very-Low-Density Lipoprotein
